# Differential Diagnosis Using MRI of Canine Intestinal Neoplasms, Including Lymphoma, Adenocarcinoma, and Inflammatory Polyp: Case Series

**DOI:** 10.1111/vru.70257

**Published:** 2026-09-17

**Authors:** Toshiyuki Tanaka, Yusuke Wada, Mizuki Tomihari, Takashi Hasegawa

**Affiliations:** ^1^ Laboratory of Veterinary Advanced Diagnosis and Treatment School of Veterinary Science Osaka Metropolitan University Osaka Japan; ^2^ Kinki Animal Medical Training Institute & Veterinary Clinic Osaka Japan; ^3^ Osaka Hospital Japan Animal Referral Medical Center Osaka Japan

**Keywords:** abdomen, apparent diffusion coefficient (ADC), computed tomography (CT), diffusion‐weighted imaging (DWI), dog, magnetic resonance imaging (MRI)

## Abstract

No studies have evaluated magnetic resonance imaging (MRI) findings of intestinal neoplasms in veterinary medicine. We hypothesized that MRI findings would differ among intestinal neoplasms in veterinary medicine. This study retrospectively assessed the MRI findings of intestinal neoplasms, including lymphomas (*n* = 6), adenocarcinomas (*n* = 2), and inflammatory polyps (*n* = 4) in dogs. The following MRI parameters were recorded: neoplasm signal intensity (SI) on T1‐weighted images (T1WI), T2‐weighted images (T2WI), contrast‐enhanced T1WI (C‐T1WI), and diffusion‐weighted images (DWI); contrast enhancement pattern on C‐T1WI; the presence of intestinal obstruction; and apparent diffusion coefficient (ADC) values. All lymphomas (6 of 6) showed hypointense SI on C‐T1WI, whereas adenocarcinomas (0 of 2) and inflammatory polyps (0 of 4) did not (*p* = 0.002). Lymphomas (5 of 6) showed homogeneous enhancement significantly more frequently than adenocarcinoma (0 of 2) and inflammatory polyps (0 of 4) (*p* = 0.02). Regarding SI of T1WI, T2WI, and qualitative DWI, and the presence of intestinal obstruction, no significant differences were observed among intestinal neoplasms. The ADC values of lymphomas (0.58 ± 0.18 × 10^−3^ mm^2^/s) were significantly lower than adenocarcinomas (1.27 ± 0.007 × 10^−3^ mm^2^/s, *p* = 0.01) and inflammatory polyps (1.56 ± 0.3 × 10^−3^ mm^2^/s, *p* = 0.0002). Lymphomas showed homogeneous low enhancement and significantly lower ADC values compared with adenocarcinomas and inflammatory polyps by MRI. These MRI findings may help differentiate intestinal lymphoma from other intestinal neoplasms.

## 1 Introduction

Imaging modalities used in veterinary medicine to investigate intestinal tumors include radiography, ultrasound, and computed tomography (CT). Recent studies have reported the use of CT in intestinal tumor differentiation [1, 2, 3, 4, 5]. However, CT of the intestine has some limitations. First, CT findings of intestinal tumors overlap across different types of tumors [5]. Second, CT has lower soft tissue contrast resolution in soft tissues and cannot effectively distinguish the layers of the bowel wall [6].

Because magnetic resonance imaging (MRI) allows for the acquisition of multi‐parameter images, it can reduce radiation exposure from CT and the use of iodinated contrast agents [7]. MRI provides excellent soft tissue contrast, detailed anatomical information, and information on lesions on hemorrhage, necrosis, edema, cystic and myxoid degeneration, and fibrosis [7, 8]. With recent advances in MRI technology, including high‐resolution three‐dimensional (3D) gradient‐recalled echo (GRE) sequences and diffusion‐weighted imaging (DWI), MRI provides improved diagnostic accuracy [9, 10, 11]. DWI has the potential to assess tissues at a cellular basis and can detect treatment response earlier than other anatomical measures [12]. MRI is used for the differential diagnosis of gastrointestinal tumors in human medicine [13, 14, 15]. MRI is the standard modality for local staging of rectal cancer, including both primary (preoperative) staging and restaging [14], as CT is unreliable for local T (tumor) and N (node) staging [14].

To the best of our knowledge, no studies have evaluated MRI findings of intestinal neoplasms in veterinary medicine. Therefore, the indications, imaging findings, and diagnostic utility of intestinal MRI are not well established. We hypothesized that intestinal MRI would reveal findings useful for distinguishing between canine intestinal neoplasms. This study reviews MRI findings in canine intestinal neoplasms and evaluates them according to tumor type, including lymphoma, adenocarcinoma, and inflammatory polyp.

## 2 Materials and Methods

This study was a retrospective case series. Owners of dogs included in this study provided their informed consent for the diagnostic procedures, treatment, and use of clinical data, such as medical history, imaging studies, and histopathological findings for research and publication purposes. As all diagnostic studies and initiated treatments were part of daily clinical activities, this study did not require submission to the local ethics and welfare committee. All dogs who underwent abdominal MRI at our institution between 2017 and 2024 were enrolled in the current study. Inclusion criteria consisted of the presence of a concurrent histopathologically diagnosed intestinal neoplasm and identification of the tissue biopsy site. The exclusion criterion was the presence of a concurrent unrelated tumor type outside of the intestinal lesion.

All dogs induced apnea by general anesthesia and were placed in dorsal recumbency. The respiratory frequency was 20 breaths per minute by mechanical ventilation. Breath‐hold was achieved using a stop ventilator. All dogs underwent MRI using a 1.5 T system (Brivo MR355; GE Healthcare Japan, Tokyo, Japan) with an anterior array express coil combined with the integrated posterior array, using an eight‐channel receiver system.

Table 1 summarizes image acquisition parameters of intestinal MRI. To assess intestinal neoplasms, including all in the lymphoma group (6 of 6), all in the adenocarcinoma group (2 of 2) and two in the inflammatory polyp group (2 of 4), MRI examinations included the following two sequences: (1) axial, T1‐weighted images (T1WI) with a breath‐hold in‐phase, opposed‐phase spoiled gradient echo (repetition time [TR]: 280 ms, echo time [TE]: 2.2/4.2 ms, flip angle [FA]: 85°, field of view [FOV]: 14–20 cm × 14–20 cm, matrix size: 160 × 160, number of excitation [NEX]: 1, slice thickness: 3 mm, interval: 0.6 mm) with no array spatial sensitivity encoding technique (ASSET); (2) axial, fat‐saturated, fast‐recovery, fast‐spin echo (FRFSE) T2‐weighted images (T2WI) with respiratory trigger (TR: 6000 ms, TE: 100 ms, FOV: 14–20 cm × 14–20 cm, matrix size: 192 × 160, NEX: 6, slice thickness: 3 mm, interval: 0.6 mm), with no ASSET.

**TABLE 1:** Image acquisition parameters of intestinal magnetic resonance imaging (MRI).

Acquisition parameters	Scan sequence				
	T1WI	T2WI	T1WI	T2WI	DWI
Respiratory control	Breath‐hold	Respiratory trigger	No	No	Respiratory trigger
Scanning sequence	In‐/Opposed‐phase SPGR	Fat‐saturated FRFSE	SE	FSE	EPI
Acquisition orientation	Axial	Axial	Sagittal or dorsal	Sagittal or dorsal	Axial
TR (ms)	280	6000	350	4600	9000
TE (ms)	2.2/4.2	100	13	120	86
FA (degree)	85	90	90	90	90
FOV (cm)	14–20 × 14–20	14–20 × 14–20	14–18 × 14–18	14–18 × 14–18	14–20 × 14–20
Matrix size	160 × 160	192 × 160	320 × 320	480 × 480	64 × 64
NEX	1	6	4	3	10
Slice thickness (mm)	3	3	2.5	2.5	3
Interval (mm)	0.6	0.6	0.5	0.5	0.6
*b* value (s/mm^2^)	N/A	N/A	N/A	N/A	600
Parallel imaging (ASSET)	Off	Off	Off	Off	On
Case group	Acquisition number of each neoplasm				
Lymphoma (*N* = 6)	6	6	0	0	6
Adenocarcinoma (*N* = 2)	2	2	0	0	2
Inflammatory polyp (*N* = 4)	2	2	2	2	4

Abbreviations: ASSET, array spatial sensitivity encoding technique; DWI, diffusion‐weighted images; EPI, echo planar imaging; FA, flip angle; FOV, field of view; FRFSE, fast‐recovery, fast‐spin echo; FSE, fast‐spin echo; NEX, number of excitation; SE, spin‐echo; SPGR, spoiled gradient echo; T1WI, T1‐weighted images; T2WI, T2‐weighted images; TE, echo time; TR, repetition time.

For the remaining two cases in the inflammatory polyp group (2 of 4), the following two sequences using standard orthogonal sections were performed: (3) sagittal or dorsal, spin‐echo (SE) T1WI without breath‐hold (TR: 350 ms, TE: 13 ms, FA: 90°, FOV: 14–18 cm × 14–18 cm, matrix: 320 × 320, NEX: 4, slice thickness: 2.5 mm, interval: 0.5 mm) with no ASSET; (4) sagittal or dorsal, fast‐spin echo T2WI without breath‐hold (TR: 4600 ms, TE: 120 ms, FOV: 14–18 cm × 14–18 cm, matrix size: 480 × 480, NEX: 3, slice thickness: 2.5 mm, interval: 0.5 mm) with no ASSET.

For all intestinal neoplasms, DWI used the following sequence: (5) axial, DWI with respiratory trigger (TR: 9000 ms, TE: 86 ms, FOV: 14–20 cm × 14–20 cm, matrix size: 64 × 64, *b* value: 600 s/mm^2^, NEX: 10, slice thickness: 3 mm, interval: 0.6 mm) with ASSET. Diffusion‐weighted gradients were applied in three directions (*x*, *y*, and *z*). For the contrast‐enhanced T1WI (C‐T1WI), the same acquisition parameters used for the precontrast T1WI were applied. C‐T1WI was performed 3 min after administration of gadoteridol 0.2 mL/kg (ProHance; Bracco Japan, Tokyo, Japan).

All images were evaluated on a workstation using commercially available DICOM image viewing software (OsiriX 13.0.1, 64‐bit, Pixmeo, Switzerland) by two veterinarians with at least 12 years of experience as veterinary radiologists (T.T. and Y.W.). Observers were aware of the types of neoplasms included in this study. However, they were blinded to the diagnosis of each individual case at the time of image review. To avoid potential bias, all images were assessed in random order in two different sessions, with at least a 2‐week interval between each session. MRI findings were recorded by consensus.

Qualitative MRI data were analyzed, and the following parameters were noted: MRI signal intensities (SIs) of the neoplasm on T2WI (hypointense, isointense, or hyperintense), SI of T1WI (isointense or hyperintense), SI of C‐T1WI (non‐contrast‐enhanced or contrast‐enhanced), neoplasm enhancement (homogeneous, heterogeneous), SI of qualitative DWI (isointense or hyperintense), intestinal obstruction (presence or absence). SI of the neoplasm on T2WI, T1WI, and qualitative DWI were qualitatively assessed between the neoplasm and intestinal parenchyma of the other intestine. SI of qualitative DWI was evaluated on high *b* value images (*b* = 600 s/mm^2^). SI of the neoplasm on C‐T1WI was subjectively compared with the SI of T1WI. When SI on C‐T1WI was unchanged compared with that on T1WI, it was defined as non‐contrast‐enhanced. The SI of the neoplasm on T2WI, T1WI, qualitative DWI, and C‐T1WI was assessed, excluding presumptive necrotic areas. Presumptive necrosis is defined as an area of high SI on T2WI without enhancement on postcontrast T1WI [16, 17, 18]. A homogeneous enhancement pattern was defined as a uniform enhancement throughout the intestinal neoplasm [19]. In the duodenum and jejunum, intestinal obstruction was defined as dilatation of the proximal loops with collapse of the distal loops [20]. In the colon, obstruction was defined as severe dilatation of the proximal colon with fecal accumulation [21].

Quantitative MRI data were analyzed for the apparent diffusion coefficient (ADC) values of each intestinal neoplasm. The quantitative ADC color map was generated from qualitative DWI images (*b* = 600) to visualize the ADC distribution using Functool ver 7.4.03 (GE Healthcare). ADC values were measured using multiple regions of interest (ROIs), avoiding cystic or necrotic areas, and averaged to yield a representative ADC for each neoplasm.

## 3 Statistical Analyses

Statistical analyses were performed using commercially available software (R version 2.12.1; R Foundation for Statistical Computing, Vienna, Austria) by T.T. Data normality was assessed using the Shapiro–Wilk test, indicating that parametric testing was required. Each intestinal neoplasm was categorized into three groups on the basis of cytological or histopathological diagnosis, including lymphoma, adenocarcinoma, and inflammatory polyp. For exploratory purposes, differences in qualitative parameters among each group were evaluated using Fisher's exact test. ADC values of each intestinal neoplasm were compared using ANOVA and Tukey–Kramer post hoc test. A *p* value of less than 0.05 was considered statistically significant.

## 4 Results

A total of 12 dogs were included in this study. Intestinal neoplasm diagnoses included lymphoma (*n* = 6), adenocarcinoma (*n* = 2), and inflammatory polyp (*n* = 4). Lymphoma was diagnosed at the duodenum (2 of 6), jejunum (3 of 6), and rectum (1 of 6). Adenocarcinoma was diagnosed at the jejunum (1 of 2) and ileum (1 of 2). Inflammatory polyps were diagnosed at the rectum (4 of 4). In lymphoma, three of six intestinal lesions were sampled by FNA, and three of six lesions were sampled by endoscopy. In adenocarcinoma, one of two intestinal lesions was sampled by FNA, and one of two lesions was removed surgically. In inflammatory polyp, all lesions were sampled by endoscopy. Intestinal neoplasms were diagnosed by cytology or histopathology.

The lymphoma group consisted of three neutered and two intact male dogs and one spayed female dog. The mean (±SD) age of dogs in the lymphoma group was 9 ± 2.8 years. The dog breeds included one Dachshund, one French Bulldog, two Pugs, one Yorkshire Terrier, and one Shiba. All cases in the lymphoma group (6 of 6) were diagnosed as high‐grade lymphoma because medium‐ to large‐sized lymphocytes were observed. In two dogs, mesenteric lymph nodes and iliac lymph nodes, including the bilateral internal iliac, bilateral medial iliac, and sacral lymph nodes, were enlarged. The adenocarcinoma group consisted of two spayed female dogs. The mean (±SD) age of dogs in the adenocarcinoma group was 10 ± 4.2 years. The dog breeds included one Dachshund and one Toy Poodle. No findings suggestive of peritoneal dissemination or enlarged lymph nodes were observed in the abdomen. The inflammatory polyp group consisted of two male dogs (one neutered and one intact) and two spayed female dogs. The mean (±SD) age of dogs in the inflammatory polyp group was 10 ± 1.2 years. The dogs included four Dachshunds. No findings suggestive of peritoneal dissemination or enlarged lymph nodes were observed in the abdomen.

Evaluation of the SI of C‐T1WI classified all in the lymphoma group (6 of 6) as hypointense, but none in the adenocarcinoma (0 of 2) and inflammatory polyp (0 of 4) groups. Lymphomas were significantly more likely to show non‐contrast‐enhanced SI than adenocarcinomas and inflammatory polyps (*p* = 0.002). Regarding lesion enhancement, five in the lymphoma group (5 of 6), none in the adenocarcinoma group (0 of 2), and none in the inflammatory polyp group (0 of 4) were classified as homogeneous. Lymphomas showed significant homogeneous enhancement compared to adenocarcinomas and inflammatory polyps (*p* = 0.02). No significant differences among intestinal neoplasms were observed in SI of T1WI, T2WI, or qualitative DWI, or in the presence of intestinal obstruction. Table 2 summarizes comparisons between neoplasm types and their qualitative MRI features.

**TABLE 2:** Qualitative computed tomography (CT) features of each intestinal neoplasm.

		Lymphoma	Adenocarcinoma	Inflammatory polyp	*p*
		*N* = 6	*N* = 2	*N* = 4	
T1WI	Hyper	0 (0%)	0 (0%)	0 (0%)	1
	Iso	6 (100%)	2 (100%)	4 (100%)	
T2WI	Hyper	1 (17%)	2 (100%)	3 (75%)	0.28
	Iso	3 (50%)	0 (0%)	1 (25%)	
	Hypo	2 (33%)	0 (0%)	0 (0%)	
CE‐T1WI	CE	0 (0%)	2 (100%)	4 (100%)	0.002
	NCE	6 (100%)	0 (0%)	0 (0%)	
Enhancement	Home	5 (83%)	0 (0%)	0 (0%)	0.02
	Hetero	1 (17%)	2 (100%)	4 (100%)	
DWI	Hyper	6 (100%)	1 (50%)	4 (100%)	0.17
	Iso	0 (0%)	1 (50%)	0 (0%)	
Obstruction	Presence	1 (17%)	2 (100%)	0 (0%)	0.06
	Absence	5 (83%)	0 (0%)	4 (100%)	

Abbreviations: CE, contrast‐enhanced; CE‐T1WI, contrast‐enhanced T1WI; DWI, diffusion‐weighted images; Enhancement, neoplasm enhancement; NCE, non‐contrast‐enhanced; Obstruction, intestinal obstruction; T1WI, T1‐weighted Images; T2WI, T2‐weighted Images.

**FIGURE 1:**
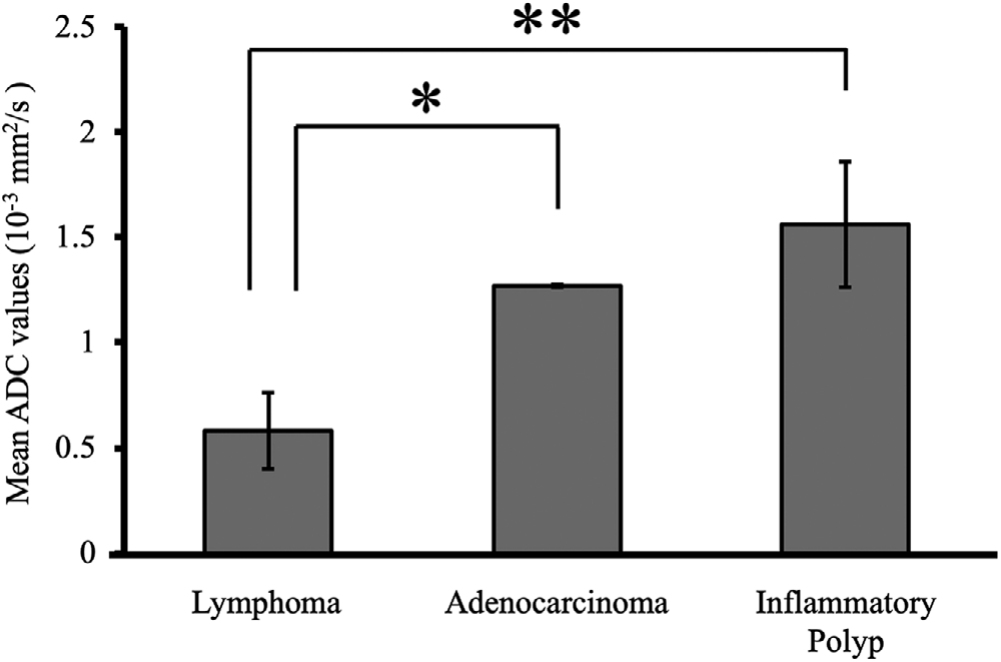
Mean ADC values of each intestinal neoplasm. ADC values of lymphomas were significantly lower than adenocarcinomas (*p* = 0.01) and inflammatory polyps (*p* = 0.0002). * and ** indicate *p* < 0.05 and *p* < 0.01, respectively. ADC, apparent diffusion coefficient.

**FIGURE 2:**
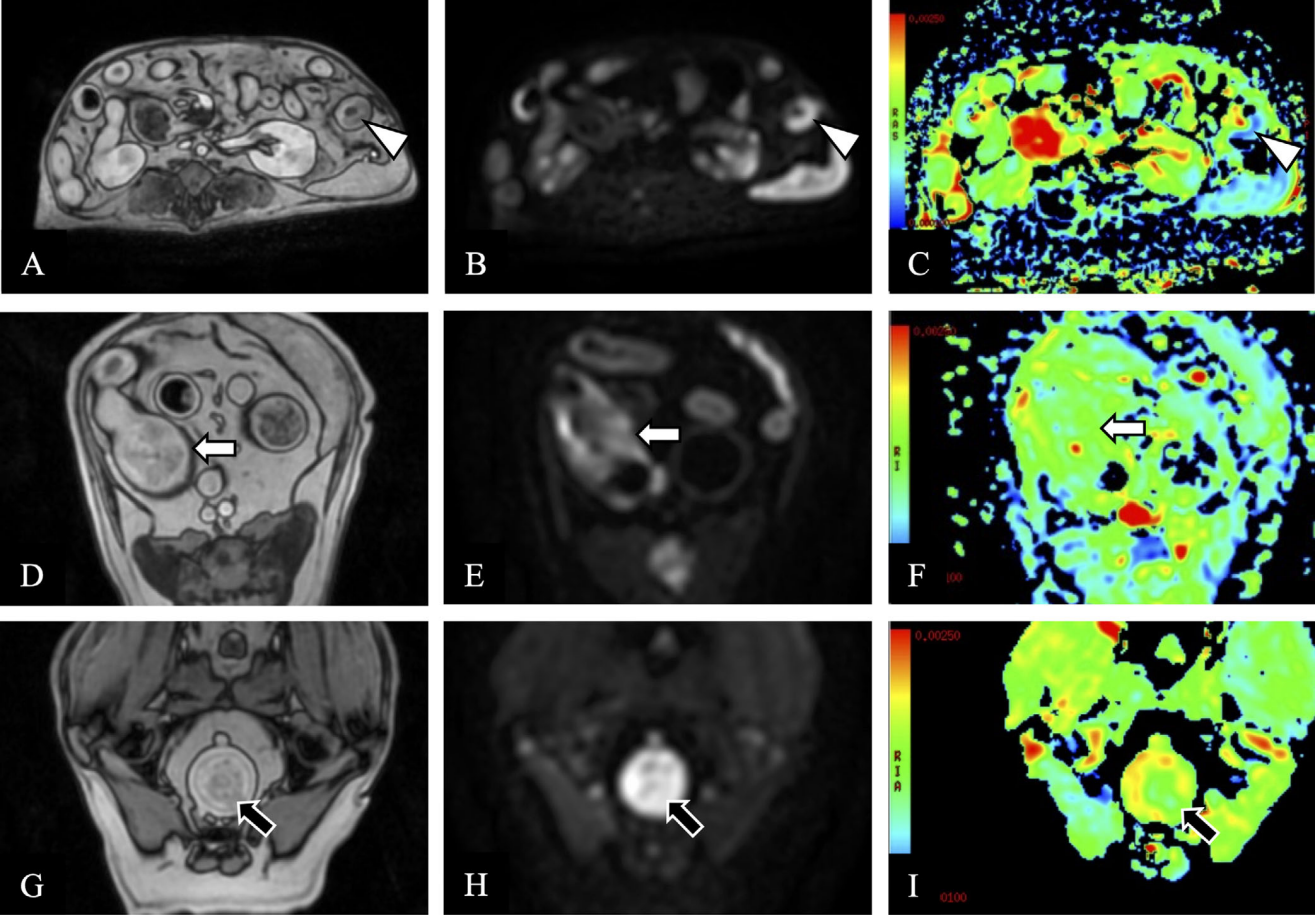
Representative MRI images of a lymphoma on opposed‐phase contrast‐enhanced T1WI (C‐T1WI) (A), DWI (B), and ADC map (C). Representative MRI images of an adenocarcinoma on opposed‐phase C‐T1WI (D), DWI (E), and ADC map (F). Representative MRI images of an inflammatory polyp on opposed‐phase C‐T1WI (G), DWI (H), and ADC map (I). The lymphoma (arrowhead) showed homogeneous and hypointense enhancement (A). The lymphoma showed hyperintense SI on DWI (B) and hypointense SI on the ADC map (C). The adenocarcinoma (arrow) and inflammatory polyp (black arrow) showed heterogeneous and hyperintense enhancement (D, G). The adenocarcinoma and inflammatory polyp showed hyperintensity on DWI (E, H) and on the ADC map (F, I).

In lymphoma, the difference in ADC distribution was visually apparent. Mean ADC values were 0.58 ± 0.18, 1.27 ± 0.007, and 1.56 ± 0.3 × 10^−3^ mm^2^/s for lymphomas, adenocarcinomas, and inflammatory polyps, respectively. The mean ADC value for lymphoma was significantly lower than adenocarcinoma (*p* = 0.01) and inflammatory polyp (*p* = 0.0002). Figure 1 shows the mean ADC values of each intestinal neoplasm. Representative images of each neoplasm are shown in Figure 2.

## 5 Discussion

In this study, lymphomas showed homogeneous low enhancement compared to adenocarcinomas and inflammatory polyps. Similarly, on CT, lymphomas are reported to demonstrate homogeneous low enhancement compared to adenocarcinomas [1, 22]. Our results indicate significantly lower ADC values in lymphomas, despite no significant differences observed in SI of qualitative DWI among intestinal neoplasms. Diffusion differences may be assessed using qualitative DWI or quantitative ADC maps [23]. DWI reflects the macroscopic mobility of water molecules in both intra‐ and extracellular spaces [24]. Restricted diffusion is characterized by high SI on DWI, whereas unrestricted diffusion results in low SI [25]. On ADC maps, restricted diffusion corresponds to lower ADC values, while unrestricted diffusion corresponds to higher ADC values [25]. As DWI is influenced by the SI of T2WI, tissues with high SI on T2WI may also appear hyperintense on DWI even in the absence of true diffusion restriction, a phenomenon known as T2 shine‐through [26]. This potential confounding effect may be assessed by referring to the ADC map, which provides a quantitative representation of diffusion‐weighted SIs [27]. In this study, 100% of adenocarcinomas and 75% of inflammatory polyps showed hyperintensity on T2WI. Therefore, SI of qualitative DWI did not differ significantly among intestinal neoplasms, potentially due to T2 shine‐through, despite differences observed in ADC values.

Lymphoma is a highly cellular tumor that restricts water diffusion, resulting in lower ADC values [28, 29]. In veterinary medicine, ADC values are reported as useful for differentiating lymphoma in the nasopharynx, nasal cavity, and intracranial region [30, 31, 32]. In humans, ADC values are also useful in differentiating gastric adenocarcinoma from lymphoma [29]. Our results suggest that ADC values may be useful in differentiating intestinal lymphoma. In humans, high‐grade lymphomas show significantly lower median ADC values compared to low‐grade lymphomas [33]. Although several studies report restricted diffusion in both lymphomatous tissue and inflamed bowel wall [34, 35], our study included no low‐grade lymphomas for comparison. Thus, differences in ADC values between high‐ and low‐grade lymphomas require future evaluation.

On CT, inflammatory polyps show heterogeneous and radial enhancement [4]. In this study, although inflammatory polyps showed heterogeneous enhancement on MRI, they did not show radial enhancement. In MRI, small lesions are mixed with the surrounding tissue in thick‐slice images by partial volume effects [36]. We hypothesize that slice thickness may contribute to differences between MRI and CT findings. In this study, MRI was performed with a slice thickness of 2.5–3 mm and an interval of 0.5–0.6 mm, whereas CT images were evaluated using 2 mm reconstruction [4]. Therefore, the findings may be affected by partial volume effects.

Adenocarcinomas arise from intestinal glands located within the mucosal epithelium adjacent to the small intestinal lumen [37]. Due to their origin, adenocarcinomas often exhibit endophytic growth, potentially resulting in obstruction of the small intestine [1]. In this study, no significant differences were observed in the presence of obstruction among tumor types, which is inconsistent with previous reports [5]. In 19% of adenocarcinomas, no obstruction is observed [38]. Intestinal adenocarcinomas vary widely, ranging from those causing severe obstruction to those without obstruction due to annular constrictive nature [38].

MRI is sensitive to motion, and respiratory motion is a major source of image degradation in abdominal imaging [39, 40, 41, 42]. Especially, T2WI and DWI are sensitive to motion [43]. Respiratory control techniques, including breath‐hold and respiratory‐triggered acquisitions, are commonly used in abdominal MRI [42]. In abdominal MRI, the breath‐hold technique is used for T1WI [44]. Although respiratory‐triggered imaging increases acquisition time because data are acquired in synchrony with the respiratory cycle, respiratory‐triggered imaging can achieve a high SNR with a sharp margin of the anatomic structures [42, 45]. In this study, two cases of inflammatory polyps involving the rectum were not acquired with respiratory control during T1WI and T2WI. Organs in the upper abdomen such as the liver, kidneys, and spleen move significantly with respiration [46]. Because the pelvic cavity is less susceptible to the effects of respiration, respiratory control may not be necessary for imaging of the rectum. Further study is needed to determine the appropriate acquisition parameters of intestinal MRI.

MRI provides excellent soft‐tissue contrast. In humans, MRI is a powerful imaging modality for gastrointestinal malignancy assessment [47]. The diagnostic accuracy of MRI for tumor‐node staging of gastric cancer is comparable to that of CT, suggesting that MRI may be a suitable alternative imaging strategy [15]. However, for systemic staging, C‐T1WI of the chest, abdomen, and pelvis remains the modality of choice according to the National Comprehensive Cancer Network (NCCN) guidelines [48]. In dogs, CT has a higher sensitivity for detecting metastases than radiography [49]. CT enables evaluation not only of the primary lesion but also of distant metastases by extending the scan range. Therefore, CT is a more frequent selection for metastatic disease evaluation for a suspected neoplasm. In veterinary medicine, MRI may be useful for detailed local evaluation of lesions by providing information such as ADC values, which cannot be obtained with CT.

This study has some limitations. First, only a small cohort of dogs with intestinal neoplasms was evaluated. In particular, there were limited cases of adenocarcinoma. Therefore, the small sample size may result in a potential Type II error. Further study about MRI findings among each neoplasm is needed with a large sample size. Second, although intestinal neoplasms occurred at several locations, including the duodenum, jejunum, ileum, and rectum, no site‐specific comparative analysis was performed. Especially, all inflammatory polyps were located in the rectum. Therefore, MRI findings of inflammatory polyps may be influenced not only by tumor type but also by anatomical location. Third, MRI sequence availability among inflammatory polyp cases varied, with variability in T2WI and T1WI. MRI sequence differences may have affected the SI comparisons for the inflammatory polyp cases. Fourth, neoplasm position varied due to intestinal peristalsis, resulting in differences in image plane across sequences, which may reduce the accuracy of SI comparisons on the same slice.

In conclusion, lymphomas showed homogeneous low enhancement and low ADC values compared with adenocarcinomas and inflammatory polyps on MRI. Intestinal MRI may have the potential to detect lymphoma.

## Author Contributions

Conception and design: Toshiyuki Tanaka. Acquisition of data: Toshiyuki Tanaka. Analysis and interpretation of data: Toshiyuki Tanaka and Yusuke Wada. Drafting the article: Toshiyuki Tanaka. Revising article for intellectual content: Yusuke Wada, Mizuki Tomihari, and Takashi Hasegawa. Final approval of the completed article: Toshiyuki Tanaka, Yusuke Wada, Mizuki Tomihari, and Takashi Hasegawa. Agreement to be accountable for all aspects of the work in ensuring that questions related to the accuracy or integrity of any part of the work are appropriately investigated and resolved: Toshiyuki Tanaka, Yusuke Wada, Mizuki Tomihari, and Takashi Hasegawa.

## Funding

The authors have nothing to report.

## Disclosure

This manuscript has not been published and is not under consideration for publication elsewhere. This case series has been reported in line with the PROCESS Guideline.

## Ethics Statement

The pet owners described in this study provided informed consent for the diagnostic procedures, treatment, and use of clinical data such as medical history, imaging studies, and histopathological findings for research and publication purposes. As each diagnostic study and initiated treatment were part of daily clinical activities, the study did not reach the threshold for submission to the local ethics and welfare committee.

## Conflicts of Interest

The authors declare no conflicts of interest.

## Data Availability

Data supporting the findings of this study are available from the first author, T.T., upon reasonable request.
